# Causes of death and associated conditions (Codac) – a utilitarian approach to the classification of perinatal deaths

**DOI:** 10.1186/1471-2393-9-22

**Published:** 2009-06-10

**Authors:** J Frederik Frøen, Halit Pinar, Vicki Flenady, Safiah Bahrin, Adrian Charles, Lawrence Chauke, Katie Day, Charles W Duke, Fabio Facchinetti, Ruth C Fretts, Glenn Gardener, Kristen Gilshenan, Sanne J Gordijn, Adrienne Gordon, Grace Guyon, Catherine Harrison, Rachel Koshy, Robert C Pattinson, Karin Petersson, Laurie Russell, Eli Saastad, Gordon CS Smith, Rozbeh Torabi

**Affiliations:** 1 Department of Genes and Environment, Division of Epidemiology, Norwegian Institute of Public Health, P.O. Box 4404 Nydalen, N-0403 Oslo, Norway; 2 Department of Obstetrics, Gynecology and Reproductive Biology, Harvard Medical School, Boston, MA, USA; 3 Department of Pathology and Laboratory Medicine, Brown University Medical School, Providence, RI, USA; 4 Department of Obstetrics and Gynaecology, University of Queensland, Mater Mothers' Hospital, Brisbane, Australia; 5 Mater Mothers' Research Centre, Mater Health Services, Brisbane, Australia; 6 Division of Family Health Development, Ministry of Health Malaysia, Putrajaya, Malaysia; 7 Department of Paediatric and Perinatal Pathology, King Edward Memorial Hospital, Perth, Australia; 8 Department of Gynaecology and Obstetrics, Kalafong Academic Hospital, Pretoria, South Africa; 9 National Center on Birth Defects and Developmental Disabilities, Centers for Disease Control and Prevention, Atlanta, GA, USA; 10 Unit of Psychobiology of Reproduction-UCADH, Mother-Infant Department, Università di Modena e Reggio Emilia, Modena, Italy; 11 Department of Obstetrics and Gynaecology, University Medical Centre Groningen, University of Groningen, Groningen, The Netherlands; 12 Department of Neonatal Medicine, Royal Prince Alfred Hospital, Sydney, Australia; 13 Alberta Perinatal Health Program, Edmonton, Canada; 14 Telethon Institute for Child Health Research, Centre for Child Health Research, The University of Western Australia, Subiaco, Australia; 15 Department of Obstetrics and Gynaecology, University of Pretoria School of Medicine, Pretoria, South Africa; 16 Department of Obstetrics, Karolinska University Hospital, Stockholm, Sweden; 17 Division of Anatomical Pathology, Department of Laboratory Medicine & Pathology, University of Alberta Hospitals, University of Alberta, Edmonton, Canada; 18 Department of Midwifery, Faculty of Nursing Education, Akershus University College, Lillestrøm, Norway; 19 Department of Obstetrics & Gynaecology, School of Clinical Medicine, University of Cambridge, Cambridge, UK

## Abstract

A carefully classified dataset of perinatal mortality will retain the most significant information on the causes of death. Such information is needed for health care policy development, surveillance and international comparisons, clinical services and research. For comparability purposes, we propose a classification system that could serve all these needs, and be applicable in both developing and developed countries. It is developed to adhere to basic concepts of underlying cause in the International Classification of Diseases (ICD), although gaps in ICD prevent classification of perinatal deaths solely on existing ICD codes.

We tested the Causes of Death and Associated Conditions (Codac) classification for perinatal deaths in seven populations, including two developing country settings. We identified areas of potential improvements in the ability to retain existing information, ease of use and inter-rater agreement. After revisions to address these issues we propose Version II of Codac with detailed coding instructions.

The ten main categories of Codac consist of three key contributors to global perinatal mortality (intrapartum events, infections and congenital anomalies), two crucial aspects of perinatal mortality (unknown causes of death and termination of pregnancy), a clear distinction of conditions relevant only to the neonatal period and the remaining conditions are arranged in the four anatomical compartments (fetal, cord, placental and maternal).

For more detail there are 94 subcategories, further specified in 577 categories in the full version. Codac is designed to accommodate both the main cause of death as well as two associated conditions. We suggest reporting not only the main cause of death, but also the associated relevant conditions so that scenarios of combined conditions and events are captured.

The appropriately applied Codac system promises to better manage information on causes of perinatal deaths, the conditions associated with them, and the most common clinical scenarios for future study and comparisons.

## Background

### A classification and its purpose

Classification is described as a passive construct systematically arranging similar entities with *established criteria *or *differing characteristics*[1,2]. The purpose of classification, however, is information management: including information capture, storage, and retrieval. Classification of perinatal deaths is thus primarily *the systematic arrangement of deaths in categories based on information known about them to aid in the process of information management*.

This information is vital for many purposes, including health care policy development, surveillance and international comparisons, clinical services and research, and it is crucial that a classification is useful for all these aims. Incompatible classifications for pathologists, obstetricians and researchers, hinder the efforts to improve the value and quality of perinatal pathology services, obstetric health care and research. All need answers as to why deaths occur, but the level of detail required differs. While 98% of perinatal deaths occur in developing countries[3,4], most research on prevention takes place in developed countries. To avoid widening the knowledge gap, a classification should be useful for all populations. In a global setting with almost 50 million unregistered live newborns and even less reporting of stillbirths, there is currently insufficient data collected on causes of death either to guide stillbirth prevention programs or to provide accountability and evidence of effect of such programs[5]. There is a need to scale up efforts to collect data on causes of death in stillbirths in developing countries. No classification system compensates for missing data and misclassifications biased towards the most easily observable conditions that may not be related to the cause of death.

A viable classification system of perinatal deaths that can be applied to existing health information systems should incorporate, or at least relate conceptually, to the International Classification of Diseases (ICD) system, serving as an information management tool for registered conditions. Identifying the underlying cause is the key concept in the cause of death (COD) in ICD[6], and a classification system must accomplish this goal as well as retain information on non-lethal conditions representing significant associated conditions. Yet, in classifying perinatal deaths, in particular stillbirths, the ICD has shortcomings. By not consistently identifying the fetus (with its cord, placenta and membranes) as an individual entity to assign codes to, there are many missing codes e.g. for the many significant placental lesions. Thus, a comprehensive classification system cannot be based on converting ICD codes alone, but must supplement ICD and serve to inform steps towards the expected revision of ICD in 2013.

The value of a classification system is not based solely on its simplicity, stringently defined and segregated categories, or inter-rater agreement and reproducibility alone. This would favor classifications that include as little information as possible. A utilitarian approach to classification would identify most clinically distinct categories of deaths, providing simplicity and reproducibility in its basic categories yet retaining important detail by extensive subcategorization or layers.

### Selecting the right information

A classification system will most consistently provide the results it was designed for, and most systems are designed to identify a few specific groups of interest. Accordingly, information managed by typical perinatal mortality classifications is often very restricted. As reviewed elsewhere[7], classifications broadly manage information in two partly overlapping groups: Some include categories best suited for epidemiology and health care planning purposes, including risk factors such as small for gestational age (SGA)[8] and twin pregnancy[9], without (or with questionable) claims to represent COD. Others aspire to manage information on COD, often focusing on specific clinical groups or categories related to biomedical research questions [10-12]. There is also diversity in the preferred source of information; encompassing to various degrees clinical obstetric diagnoses, pathology reports, full medical charts, or simply routinely provided ICD-codes. In testing contemporary classifications of stillbirths, Flenady et al found inconsistent approaches to the main categories of stillbirth, making datasets and classifications difficult to compare[7]. These problems are especially noteworthy regarding the classification of placental causes of death[13]. But despite apparently conflicting priorities, all categories are easily combined into one utilitarian system managing the most significant information irrespective of source or intended use.

In most clinical and research settings, in ICD, in death certificates and cause of death registries the COD is the main focus, based on the concept of underlying causal conditions. Associated conditions as, for example, SGA and twin pregnancy may be less valued. When relevant, these can often be deduced from the specified COD, but not vice versa. E.g. SGA engulfs just about every lethal chronic condition in fetal life, and when twinning is truly part of the causation, it will be captured by COD as entangled cords, twin-to-twin transfusion, etc.

The best understanding of COD is provided by the well educated health care micro-system surrounding the woman, which may include her clinical care providers, microbiologist, geneticist and perinatal pathologist. A multidisciplinary audit group reviewing deaths at her birthing institution remains the "Gold Standard" for classification of perinatal deaths, and the backbone for improvement of care through feedback[14]. They collect all relevant information for classification and establish diagnoses, and have the narrative – the sequence and relative significance of events – to understanding why the death occurred. A classification should preserve and manage information on both individual conditions and their relative importance from the narrative.

Availability and appropriateness of care is sometimes an essential part of the narrative, and this information is needed to guide health care policies for the prevention of deaths. To preserve information about an intrapartum death, "unavailable obstetric care" may be more important than "malpresentation" in developing countries, but sub-optimal care is also frequently reported in perinatal deaths in developed countries[15], and this must be understood and such deaths prevented.

### The continuum of perinatal loss

The numerator in perinatal mortality classification is the number of individual fetuses entering the perinatal period alive, and dying before it ends. The perinatal period is defined (see table 4) by the WHO as being ≥ 500 grams, and only secondarily ≥ 22 weeks [6], as gestational age is often unknown in developing countries. Yet, in practice, gestational age has been used interchangeably with birthweight in many settings, and most legal implications are linked to gestational age [16-18]. Gestation at birth is commonly used, but should be corrected if time of intrauterine death is known. Valid arguments can be made for other limits of gestation, but for uniform reporting the limit at 500 grams/22 weeks is internationally established, and the WHO also recommend reporting data for international comparisons with the limits 1000 grams/28 weeks/35 cm. Nonetheless, communities who use lower limits should register and classify all their cases and causes of death accordingly, and many would favor also registering late neonatal deaths until the 28^th ^postnatal day (rather than the 7^th ^postnatal day) to capture more deaths with origins in perinatal events.

It should be noted that mean birthweight of stillbirths is significantly below 500 g at 22 completed weeks. Communities using the birthweight criterion will underestimate their stillbirth rate compared with regions using gestational age. In a population-based material with close to universal ultrasound-dating[19], 10.4% of stillbirths (> 22 weeks) would be unreported if the birthweight criterion was used.

In addition to variations in definitions, incomplete registrations, and differences in registration preferences and cultures, two classification issues hamper comparisons of stillbirth rates: "truncated" data due to terminations of pregnancy, and the "transfers" from stillbirths to neonatal deaths due to medical interventions (e.g. early delivery for hypertensive disorders). To enable interpretation of cause-specific perinatal death rates, information on both terminations and neonatal deaths must be managed together with stillbirths.

Neonatal deaths are often reported at any gestation as long as signs of life are seen. Yet, for consistency, the numerator should remain the same, and neonatal deaths only reported for the cases who have entered the perinatal period alive (500 g/22 weeks corrected post-natal age or weight/length at time of death) and thus qualifying as perinatal deaths.

No criteria can capture terminations with equal consistency. They will cause variations in perinatal mortality reports, some jurisdictions having mandatory registration of terminations among perinatal deaths[20]. When using the same criteria to register both terminations and spontaneous deaths, higher termination rates yields lower anomaly mortality rates[20] (many terminations prior to 22 weeks, not registered, would die perinatally if continued), while extending registrations to include lower gestational age criteria for terminations than for spontaneous deaths add the opposite effect (anomalies may cause death prior to perinatal life, and should not be included). With no perfect solution, the inevitable compromise should reflect the purpose of classification; capturing information on causal contributors to perinatal mortality.

## Methods

### The Codac system

Based on the requirements discussed above, we propose the Codac classification – developed to code the COD, aided by two associated conditions (AC) to preserve essentials of the narrative. We recommend having the classification file open to explore while reading the descriptions and instructions below [see Additional file 1]. The basic definitions, on which the development of Codac was based, are presented in table 4.

**Figure 1:**
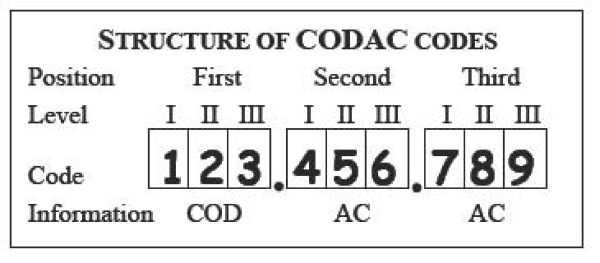
**Structure of codes in Codac**. Up to three codes can be assigned for each case, where the code in the first position represents the main cause of death (COD), while the codes in the second and third positions represent the associated conditions (AC). Within each of the three individual codes, the first digit represents level I and the main categories in Codac (table 1), while the subsequent two digits represent the subcategories of level II and III.

Codac allows up to three codes with three digits each. The main (or single) COD is coded in first position, while the AC (or secondary COD) are coded in second and third positions (figure 1). The first digit in a Codac code represents Level I with the main categories (table 1). The second and third digits represent Levels II and III where 0, indicating "Unspecified", is the default entry. In Level III categories the digit 9 indicates "Other" specified with a free text entry.

**Table 1:** Level I categories of Codac

**Categories of the primary causes of death (COD) and associated conditions (AC)**	
0	Infectious causes of death (abbrev: Infection)
	Deaths caused by infections affecting the mother, neonate or intrauterine structures and compartments directly are coded here by the causative agents as the primary COD. This includes lethal effects of infection by leading to congenital anomalies, by causing direct failure of the placenta or vital fetal/neonatal/maternal organs, or by initiating pre-viable preterm labor. The locus of the infection may be coded in subsequent positions.
1	Conditions, diseases and events specific to neonatal life (abbrev: Neonatal)
	Neonatal deaths caused by conditions or events specific to neonatal life are coded in this category as primary COD. Other COD and AC for neonatal deaths may be coded in any other relevant category.
2	Mechanics and events of parturition or its complications (abbrev: Intrapartum)
	Deaths occurring after onset of labor (intrapartum or neonatal) and where the most significant causal mechanisms were initiated by the onset, progress or complication of labor, are coded in this category as primary COD. Cases in which pre-existing conditions had reduced fetal survival potential to such an extent that mortality in normal and otherwise uncomplicated labor is significant (proportion > 0.05) if undelivered, should be coded with that condition as the primary COD with Intrapartum in a subsequent position.
3	Congenital anomalies, chromosomal anomalies and structural malformations (abbrev: Congenital anomaly)
	Deaths caused by congenital and chromosomal anomalies and structural fetal malformations, including effects of amniotic banding, are coded here as primary COD. Malformations of the placenta and cord are coded in those categories, with the exception of amniotic banding which are all coded here, irrespective of structures affected. Disruptions/deformations due to maternal uterine malformations are coded in Maternal. Congenital neoplasia is coded in Fetal.
4	Fetal conditions, diseases and events (abbrev: Fetal)
	Deaths caused by any fetal condition, disease or event (except Congenital anomaly) are coded here as primary COD. This includes those caused by placental transfer of toxins, or maternal antibodies against fetal tissues (as in alloimmunization) that does not constitute a maternal disease. The effects of maternal antibodies against her own tissues (as in anti-cardiolipin syndrome causing placental thrombosis or SS-A/SS-B antibodies causing fetal arrhythmias), should however be coded in Maternal.
5	Cord conditions, diseases and events (abbrev: Cord)
	Deaths caused by any condition, disease or event affecting the umbilical cord and its insertion are coded here as primary COD. If the same process has been shown to be present and equally significant in the fetal compartment, the primary COD should be coded there, if applicable.
6	Conditions, diseases and events of the placenta and membranes (abbrev: Placenta)
	Deaths caused by any condition, disease or event affecting the placenta and membranes are coded here as main COD. If the same process has been shown to be present and equally significant in the fetal or cord compartment, the primary COD should be coded there, if applicable.
7	Maternal conditions, diseases and events (abbrev: Maternal)
	Deaths caused by any maternal condition, disease or event, of a sufficient degree to significantly increase the risk of perinatal death are coded here as primary COD. If the same process has been shown to be present and equally significant in the fetal, cord or placental compartment, the primary COD should be coded there, if applicable.
	This category includes conditions that was unrelated to of pregnancy (as in maternal cancer), was incompatible with a viable pregnancy (as in Ehler-Danlos syndrome), was exacerbated by the normal physiology of pregnancy (as in anti-phospholipid syndrome), or was caused by uncertain mechanisms of pregnancy, and yet poses serious threats to maternal and fetal health (as in acute fatty liver of pregnancy). In exceptional cases, the category may include maternal pathology provoked by non-lethal pathophysiology of pregnancy (as in acute onset pregnancy-induced hypertensive crisis with apparently minimal placental pathology). Symptoms (as hypertension) caused by intrauterine pathologies (as placental insufficiencies) should not be coded as a COD, but may be coded in subsequent positions.
8	Unknown, unexplained and unclassifiable causes of death (abbrev: Unknown)
	Neonatal, antepartum, and deaths with unknown timing, in which no definite or probable COD has been found are coded in this category as the primary COD. Otherwise unclassifiable cases are also coded here. This category only exists for causes of death, and is replaced by Associated perinatal for AC.
9	Terminations of pregnancy (abbrev: Termination).
	All deaths caused by termination of pregnancy are coded in this category as the primary COD. This is irrespective of the indication, timing of death, or whether termination was performed by health professionals or not. It includes augmentations of labor in cases of expected unavoidable death, and also cases in which death did not occur before the completion of delivery. This category only exists for causes of death, and is replaced by Associated maternal for AC.
**Categories specific to associated conditions**	
8	Associated conditions and complications in the perinatal period (abbrev: Associated perinatal)
	AC and complications of pregnancy are coded here in the secondary or third position.
9	Associated maternal conditions and identified risk (abbrev: Associated maternal)
	AC and identified risk of the mother are coded here in the secondary or third position.

In addition to the actual Codac classification, the electronic file also enables the reporting of timing of death, the level of evidence available to the coder, and other core characteristics of the case. A brief print version based on Level I and select categories of Level II has been made for low resource settings (Simplified Codac, see table 5 and discussion below).

The ten Level I categories consist of three key contributors to global perinatal mortality: *Mechanics and events of parturition or its complications (*abbrev:*Intrapartum), Infectious causes of death (*abbrev:*Infection) *and *Congenital anomalies, chromosomal anomalies and structural malformations (*abbrev:*Congenital anomaly)*, two crucial concepts of perinatal mortality: *Unknown, unexplained and unclassifiable causes of death (*abbrev:*Unknown) *and *Terminations of pregnancy (*abbrev:*Termination)*, a clear distinction of conditions relevant only to the neonatal period: *Conditions, diseases and events specific to neonatal life (*abbrev:*Neonatal)*, and the remaining conditions are arranged in the four anatomical compartments: *Fetal conditions, diseases and events (*abbrev:*Fetal)*, *Cord conditions, diseases and events (abbrev: Cord)*, *Conditions, diseases and events of the placenta and membranes (*abbrev:*Placenta) *and *Maternal conditions, diseases and events (*abbrev:*Maternal)*.

Categories available in first position may represent a COD (table 1), while associated conditions that can never fulfill criteria for COD irrespective of the severity, are restricted to second and third position in category 8 (*Unknown *in first position) which becomes *Associated perinatal *conditions and 9 (*Termination *in first position) which becomes *Associated maternal *conditions.

### Coding the causes and associated conditions

Ten coding rules have been defined for Codac. These are summarized in table 2, and detailed below. The general understanding of causation vs. associations is well-established[21], and not discussed here. For the purpose of classification, COD and AC differ from mechanisms and pathophysiological chain of events in dying. Only COD and AC are coded in Codac, not mechanisms (table 4). For example, a lethal case of placental infarctions (code 640) initiates hypoxic-ischemic pathways which result in fetal brain injury. Infarctions may also lead to placental abruption (code 635), but not consistently, before hypoxic-ischemic brain injury develops. In the latter case, both codes 640 and 635 should be used, but in neither case should fetal brain injury be coded.

**Table 2:** Coding rules in Codac

1.	To be a COD, the condition(s combined) should have significant lethality (≥ 0.05) in the clinical setting it was observed.
2.	If no COD was found, code antepartum stillbirths and neonatal deaths as 8xx and intrapartum deaths as 29x.
3.	If two (or more) conditions could be COD, select the most significant contributor to death.
4.	If two equally significant conditions could be COD, code the first to occur if this can cause the latter (related conditions)
5.	If two equally significant conditions could be COD, code the last to occur if this cannot cause the first (unrelated conditions)
6.	If two equally significant conditions of unknown timing could be COD, code the first among codes 0 to 7 (hierarchically).
7.	If COD was infectious, code as 0xx (000 if unknown agent) and report the locus as AC in 19x, 49x, 59x, 69x or 79x.
8.	If any act to advance death was performed (termination), code as 9xx, and conditions leading to termination as AC.
9.	To be an AC, the condition(s combined) should contribute significantly in explaining the circumstances of death.
10.	Do not code any condition(s) unrelated to the causes or circumstances of death.

Both for COD and AC, the order of codes should preserve the relative significance and sequence of events. In a sequence where one condition is both necessary and sufficient to cause another condition, the underlying cause is coded as COD.

As elsewhere in medicine, the final decision as to what constitutes a perinatal COD is a subjective expert opinion, and discussions on what conditions represent COD are inevitable. To guide this discussion, we expect a COD other than "unknown" to be coded only if the single or combined conditions are mortal in a significant (> 0.05) proportion of cases (more than a tenfold relative risk in most developed countries). The answer to the question *"Is it probable that more than one in 20 cases with these conditions in this situation would die by this cause?" *should thus be *"yes" *(rule 1, table 2). When biologically plausible mechanisms or chain of events have been demonstrated, a lower relative risk of death is acceptable. E.g. fetal Kell alloimmunization may have low mortality in general but with documented anemia leading to hypoxia-ischemia and death of no other obvious etiology, it fulfils the criterion for COD.

In complex clinical situations, there may be several causal factors – or "multiple hits". When two or more conditions were causal, the coded COD does not have to fulfill the definition of COD by itself, as long as the scenario of combined conditions fulfills the requirements.

If no single or combination of associated conditions fulfills the criteria for COD, it is crucial to segregate unexplained deaths from the incompletely investigated[19]. Unexplained antepartum, neonatal, and deaths of unknown timing are classified in subcategories of code 8 *Unknown, unexplained and unclassifiable causes of death (abbrev: Unknown)*. Unexplained intrapartum deaths are coded in subcategories of code 29 *Mechanics and events of parturition or its complications (abbrev: Intrapartum) – unknown (fetal respiratory failure/asphyxia) *– parturition being the only known significant event (rule 2, table 2). Conditions that do not fulfill the criteria for COD may still be coded as an AC if eligible.

If several pathologies are present, while no direct association indicate one of them as the underlying cause of the other(s), the most significant lethal condition is coded as COD (rule 3, table 2), but if equally significant they are coded as follows:

1) If related conditions, the pathology considered to occur first is coded as the COD (due to the probability that the first caused the second) (rule 4, table 2). E.g. isolated multiple infarctions of the placenta and placental abruption. 2) For unrelated conditions, the last to occur is coded as COD (due to evident survival of the first condition) (rule 5, table 2). E.g. an infection and true knots of the cord with evidence of circulatory compromise. 3) When no indication of timing is available, the hierarchical approach described below is used (rule 6, table 2).

The assumption of mutually exclusive categories makes limited clinical sense, most cases having several contributing causes, but Codac categories are distinctive – with one significant exception: Infections are coded in two separate positions comparable to ICD: First by infectious agents (codes 0xx), and second by site of infection (codes 19x, 49x, 59x, 69x, 79x) (rule 7, table 2). Even infections by unknown agent are coded as COD by unspecified infection (code 000) with site of infection in the second position to retain the information. In neonatal congenital infections (contracted by definition in fetal life), site of the underlying infection is coded in the second position among fetal infections (codes 49x).

As previously discussed, registrations of terminations must be based on a compromise. In Codac, we recommend reporting all terminations 1) in the perinatal period, and 2) for conditions constituting a perinatal COD – starting whenever consistent registration is feasible after 12 completed weeks. Terminations in cases of inevitable death, e.g. inductions prior to 22 weeks for inevitable pregnancy loss in chorioamnionitis with preterm prelabor rupture of the membranes (PPROM), or for maternal health issues such as severe preeclampsia, should only be registered if the natural history of the disease or condition indicated that they were expected to enter the perinatal period alive. In Codac, terminations are coded in category 9 and to gain information on how terminations affect mortality rates, the reason for termination is coded both in the main and subsequent categories (rule 8, table 2).

It is crucial to only code conditions if there is evidence that these were significant factors in explaining the death. Non-compliance to this leads to skewed and excessive coding of prevalent and easily observed conditions. E.g. obesity should be coded as AC only when it is a risk factor for the COD in the actual case (rules 9 & 10, table 2).

### Hierarchical coding

In Codac Version II, Level I is organized with a hierarchical structure (starting with category 0) to provide a partial substitute when information on sequence and relative significance of conditions is lacking. In levels II and III, there is no hierarchy. The rationale for the hierarchical sequence combines 1) the perceived severity of identical findings in different categories (e.g. most infections are more likely to be indicative of a direct COD when found to be active in the fetus, and gradually less likely if found only in the cord, placenta or mother), 2) temporal proximity to death (e.g. neonatal deaths following both fetal and neonatal events are more strongly attributable to neonatal events), and 3) the most prevalent sequence of events (e.g. chromosomal abnormalities precede and may cause other fetal conditions, not vice versa).

### Testing and improvements in the Codac classification

We refined the system after initial testing, addressing improvement opportunities identified in the study testing the original version of Codac in comparison with different classification systems[7] with scoring based on how easily the right category was found, and how well existing information was retained after classification. For the performance of the original version of Codac, we refer to that publication.

In the development of the second version of Codac that we present here, we used three approaches that proved to contribute significantly in the improvement of the original version: 1) All entries of "Other" with free text were explored to identify "missing" categories that should be added. 2) All categories that received higher score in any other classification, or that received low scores for either easiness of use or the capture of information in more than 10% of cases, were revised to identify improvement opportunities. 3) All categories at Level II that were used in less than 1% of cases were considered for demotion to Level III, while Level III categories used in more than 2.5% of cases were conversely considered for promotion to Level II.

### Consistency of coding in Codac

To test inter-rater agreement of COD in Codac version II, we used 100 randomly selected cases from the material used in the study testing different classifications[7]. Each case was presented to the coders as a brief summary of clinical findings with results of pathology and other tests, if any. Significant information was often lacking (see table 6 of coding examples). The coders were given an earlier version of the draft manuscript above as instructions, but without table 6 with examples or table 2 with the summary of coding rules in Codac.

## Results

Several coders failed notably in following the three testable instructions in Codac: 1) rule 7, to code infectious causes in *Infection *(coding by the site of infection instead), 2) rule 8, to code terminations for congenital malformations in *Termination *(coded as *Congenital anomaly *instead), and 3) rule 2, to code unexplained intrapartum deaths in *Intrapartum *(coded as *Unknown *instead). Only coders 1 and 6 followed instructions in > 3/4 of cases (80 and 75%, respectively), with a Kappa value of 0.82 between them (table 3). Coder 4 failed to follow instructions in 86% of cases, and hence the low Kappa values for this coder. Coders 2, 3, 5 and 7 followed instructions in half their cases (46, 52, 60 and 52%, respectively).

**Table 3:** Agreement among coders

	**Coder 6**	**Coder 5**	**Coder 7**	**Coder 3**	**Coder 2**	**Coder 4**
**Coder 1**	0.82	0.71	0.73	0.70	0.70	0.53
**Coder 6**		0.71	0.68	0.63	0.74	0.57
**Coder 5**			0.67	0.61	0.69	0.51
**Coder 7**				0.61	0.56	0.52
**Coder 3**					0.63	0.54
**Coder 2**						0.59

Kappa values for the agreement in coding of the main cause of death category among coders. Coders are listed (left to right and top to bottom) by their falling adherence to the coding instructions of Codac. Mean adherence of coders 1 & 6 (top left) was 78% and of coders 2 & 4 (bottom right) was 30%.

**Table 4:** Definitions

**Perinatal period**
*The perinatal period commences as the birth weight passes 500 grams, or 22 completed weeks of gestation if weight is unknown, or 25 cm crown-heel length if weight and age is unknown, and it ends with the early neonatal period at 7 postnatal days.*
**Fetal death**
*Fetal death is death prior to the complete expulsion or extraction from its mother of a fetus, irrespective of the duration of pregnancy; the death is indicated by the fact that after such separation the fetus does not breathe or show any other evidence of life, such as beating of the heart, pulsation of the umbilical cord, or definite movement of voluntary muscles.*
**Stillbirth**
*A stillbirth is the birth after fetal death in the perinatal period.*
**Perinatal death**
*A perinatal death is death during the perinatal period, and includes stillbirths and early neonatal deaths.*
**Cause of death**
*A cause of death in stillbirth is an event, disease or condition of sufficient severity, magnitude and duration for death to be expected in a significant proportion of such cases in a continued pregnancy in the clinical situation it was observed.*
COD in the neonate is defined likewise by deleting the insert "*... in a continued pregnancy ...*"
**Associated condition**
*An associated condition of stillbirth is an event, disease or condition of sufficient severity, magnitude and duration to contribute in explaining the circumstances of death in a significant proportion of such cases in a continued pregnancy in the clinical situation it was observed.*
AC in the neonate is defined likewise by deleting the insert "*... in a continued pregnancy ...*"
**Mechanism of death**
*Mechanisms are biological pathways or chains of events that are initiated by an underlying cause, and consistently and irreversibly result in the same ultimate outcome when triggered by the same event.*

## Discussion

The Codac classification has benefited from being discussed and developed over an extended time period by a diverse group of experts, being independently tested in several populations by different groups of coders, and amended according to user feedback prior to publication.

### Consistency of coding and scenarios

In the traditional measure used to test classifications, the inter-rater agreement (table 3) illustrate that adherence to the coding rules of Codac is both needed, and sufficient, to obtain comparable groups for analysis. Table 2 and the panel with examples were added after the study to address the most common errors in coding. Of importance, diverse coding is the expected result when there are differing views on adequacy and relative significance of potential causes of death. No classification will resolve differences in expert opinion. Yet, when considering all three codes, the Kappa value between coders 1 and 6 for coding the condition used as the main COD by one of them, as either a COD or an AC by the other, was 0.94. Such measures are important, as many deaths are the result of either predictable "multiple hits" or typical scenarios well known in clinical practice. In the 857 cases tested in the evaluation study[7], the combination of placental infarctions and maternal hypertensive disorders represented 3% of deaths, 24% of cases with hypertension, and 22% of cases with placental infarctions. Infections by common bacteria of maternal flora and PPROM represented 2% of stillbirths, 21% of infections, and 50% of cases with PPROM. To reflect this, perinatal mortality rates by COD should be supplemented with analysis by frequent associations and scenarios provided automatically by the Codac system. At Level II (two digit codes), such scenarios with two conditions are relatively common, while scenarios of three conditions are rare.

Yet, the true test of a classification system is whether it meets the user's needs to succeed in their endeavors – in stillbirth prevention or research. There are four areas of much debate where others may perceive our choices as limitations: limits of lethality, hierarchy, definitions of categories, and complexity vs. simplicity.

### Limits of lethality

It has been suggested to define categories as lethal vs. non-lethal by unambiguous quantitative dichotomizations, e.g. fetomaternal hemorrhages > or < 40% of estimated total blood volume, respectively[22]. Although technically appealing, we believe it has questionable merits. Such arbitrary limits for individual conditions lack evidence, ignore the clinical complexity of causal factors, and might result in unwarranted differences in the defined mortality rate. E.g. placental abruption (>5% mortality[23]) is typically accepted as a COD, but the actual mortality at the suggested limit of involvement of > 30% of the placenta[22] may be as low as < 1%[23].

In most cases the narrative, clinical setting and findings documenting the chain of events will provide sufficient guidance for an expert opinion on the COD. The requirement of significant lethality in Codac should dissuade coding less severe conditions as COD, such as uncomplicated maternal diabetes type 1 or preeclampsia, isolated nuchal cord without sign of compromise, or minor single field structural congenital anomalies. Only when the narrative is missing or mechanisms are unknown, the requirement for known significant lethality plays a direct role in defining COD: e.g. uncomplicated maternal malaria (without placental pathology) is not a COD, while trisomy 21 is. When estimating such mortality rates, the complete period at risk should be carefully considered, as conditions may carry very different risks in antenatal, intrapartum and neonatal life[24]: e.g. fetuses with a diaphragmatic hernia have an antepartum mortality > 30% because of the association with other malformations, but in isolation, congenital diaphragmatic hernia only fulfils the criterions for COD in neonatal deaths, not stillbirths[25].

### Hierarchy

We have chosen not to have a strict hierarchical system that some argue will provide more consistent coding, and only offer a hierarchical system for situations in which the actual narrative has been lost. Aiming to preserve the narrative is incompatible with a strict hierarchical system where the coder is forced to select the COD from the "top of the list" although another cause was evidently a more significant contributor. Forced coding of "false" narratives when the "Gold Standard" is present, e.g. from a multi-professional audit, affects quality of data and training/motivation of users. However, in settings such as population-based surveillance, deaths may be reported with a very limited narrative (only with a prioritized main COD from death certificates), or even unsorted selections of codes (ICD or other) deemed to be of significance. Despite sub-optimal data such large databases provide riches for research and surveillance, and need systematic information management.

### Definitions of categories

The Codac system does not provide definitions of all individual categories. We have intended to reduce the consequences of this limitation by making many categories in Codac similar to ICD, and the Codac file system cross-references the equivalent ICD codes. Most gaps in ICD relate to placental and cord pathologies. Established definitions of categories such as hypercoiled or uncoiled/hypocoiled umbilical cord[26] and distal (terminal) villous hypoplasia[27] must be found elsewhere. Some conditions as e.g. "Small for gestational age" have several well established definitions and are kept in Codac as subcategories.

### Complexity vs. simplicity

In a developing country where most deaths occur in unattended home deliveries, data mainly come from verbal autopsies. These are classified by a limited number of conditions that are easily observed clinically, not the COD which is often perceived as obscure. This is valuable information for health care planning locally, and for international estimates of the burden of such conditions. However, the knowledge transfer and comparison between the developing and developed world may be limited. Although high resource setting may easily report such categories, it does not serve their own needs and such classifications cannot be further subcategorized into actual COD. A solution for developing settings may be to combine two classifications and gradually improve their data on COD as coverage for basic obstetric care and examinations improve.

The full detail of Codac will be of restricted value considering the challenges of information gathering in developing regions, and the Simplified Codac (table 5) is suggested as a tool for such settings. The Codac system reports the broad classification of perinatal deaths as well (i.e. neonatal, intrapartum and antepartum). Reported rates of COD categories will be skewed by the availability of information, related to the rate and quality of post mortem and placental examinations. Classifications with detailed categories based on extensive testing will be more prone to this effect. For better reproducibility and user friendliness, Level I has few and broad main categories, easily applicable even with little information available. Levels II and III, on the other hand, must be interpreted with care when comparing populations with different resources or preferences for post mortem examinations. The Codac system offers the opportunity to register the level of evidence and information available at the time of classification so that the value of the registered information can be estimated accordingly. The detail presented in Levels II and III may positively guide post mortem protocols by suggesting requirements to identify the COD in full detail. Level II of the COD represents the broad and significant clinical groups, and the work-up should aim to exclude or affirm their presence – at a reasonable level according to local resources[28,29].

**Table 5:** Simplified Codac.

**0) INFECTION**	02 MALARIA		
	04 SYPHILIS		
	05 GROUP B STREPTOCOCCUS (GBS)		
	06 COMMON BACTERIA OF MATERNAL FLORA (NON-GBS)		
			
**1) NEONATAL**	11 EXTREME PREMATURITY		
	13 CARDIO-RESPIRATORY		
	19 INFECTION		
			
**2) INTRAPARTUM**	23 MALPRESENTATION		
	25 PROLONGED/OBSTRUCTED OR INCOMPLETE LABOR		
	26 EXTREME PREMATURITY		
	29 UNKNOWN (FETAL RESPIRATORY FAILURE/ASPHYXIA)		
			
**3) CONGENITAL ANOMALY**	31 CENTRAL NERVOUS SYSTEM		
	32 CARDIOVASCULAR AND LYMPHATIC VESSELS		
	37 TRISOMIES		
			
**4) FETAL**	43 ALLOIMMUNIZATION		
	47 HYDROPS OF UNKNOWN ORIGIN		
			
**5) CORD**	51 KNOTS		
	52 LOOPS		
	53 ABNORMAL INSERTION		
			
**6) PLACENTA**	63 ABRUPTION		
	64 INFARCTIONS AND THROMBI		
			
**7) MATERNAL**	71 HYPERTENSIVE DISORDER		
	73 DIABETES		
	79 INFECTION		
			
**8) UNKNOWN**	81 UNKNOWN	**8) ASSOCIATED PERINATAL**	81 SMALL FOR GESTATIONAL AGE
	85 UNEXPLAINED		83 MULTIPLES
	86 UNCLASSIFIABLE		89 SUBOPTIMAL CARE
			
**9) TERMINATION**	91 FOR CONG. ANOMALY	**9) ASSOCIATED MATERNAL**	91 OBSTETRIC HISTORY
	94 FOR FETAL DISEASE		92 SMOKING
	96 FOR MATERNAL CONDITION		95 POVERTY

If only the level I code is entered, the default level II subgroup is 0 = "Unspecified or other".

**Table 6:** Coding examples

Case scenarios (selected cases used in agreement study) and coding comments by JFF:

**Case 108**. 34 yrs, G1, P0. Normal pregnancy till 41 w: Proteinuria (+3) and pregnancy induced hypertension (BP previously normal). Presented to birth clinic with contractions and rupture of membranes, non-reactive CTG, emergency CS, Apg 0-0-0. Baby girl. BW 2676 g, not IUGR. Placenta 600 g. Autopsy, PAD of placenta, screen for infections and Kleihauer neg. Unexplained death (acute intrapartal asphyxia, but unexplained why this happened).
*"Coding rules 1 and 2 apply here. No condition fulfils criterions for a COD, but labor was a significant event and it should be coded 291 as COD. I would probably have added 714 for the preeclampsia as AC, but I would have liked to know more about the blood pressure."*
**Case 207**. G7, P4. Unknown gestation (24–26?), no prenatal care. Smoke – <10 cig/day. Alcohol in pregnancy – alcoholism. History no fetal movements preceding two weeks. Presenting with mild bleeding. Placenta small, extensive infarctions with fibrosis, thickened opaque membranes.
*"The main problem here is rule 1. I'm not sure I can say what the COD was, but with the report saying both extensive infarctions and a small placenta, I would probably code 644. With this scarce report, I'm not sure her delay of 2 w without fetal movements is important, so rule 10 prevents me from coding 891 as an AC. I would add codes 921 and 931 for maternal smoking and alcoholism.*"
**Case 210**. 29 yrs, G7, P3, A3. Presented with ruptured membranes at 22 w and decreased fetal movements. SPROM × 3 days. Antibiotics antepartum and in labour. Breech. Fever. Oligohydramnios. Normal BP. History smoking during pregnancy <10 per day. PAD of placenta report chorioamnionitis, marked deciduitis, Ascending infection. Dismature, numerous hemorrhages consistent with maternal hypertension.
*"It doesn't say much about what happened around the time of death. But I read this as a death caused by an infection and rule 4 apply. I would code 000 as COD. As the site of infection I would code the placenta with acute chorioamnionitis in 691. In many cases these deaths will occur intrapartum due to extreme prematurity (code 262), but without evidence for it, I would code the lengthy PPROM as 862."*
**Case 402**. 26 yrs, G2, P1. Previous baby 3200 g term and OK. Unbooked and presented with no fetal movements for 8–10 weeks. Induced and delivered a macerated stillbirth. Cell culture from chorionic villi, Trisomy 21.
*"Little information on any actual cause of death. Yet, trisomy 21 does have an antepartum mortality > 5% even without any malformation, so according to rule 1 it should be coded 376."*
**Case 407**. 32 yrs, G4, P2, TOP 1. Uneventful pregnancy. Presented with absence of fetal movements. Antepartum fetal death at 38 weeks. 3020 g. Autopsy, PAD of the placenta and all other tests performed according to guidelines with no findings. Unexplained.
*"The case description is brief, but it states specifically that all tests were performed according to guidelines, and it should be coded as a true unexplained stillbirth – 861."*

### Future updates and amendments

New knowledge will necessitate modifications of the classification, and this system has the flexibility to accommodate most foreseeable revisions. To avoid transition problems between versions, amendments should be made with care to provide robust re-coding possibilities. The authors plan to update Codac in 2013, based on four years of experience and pending the expected release of ICD 11 to which the next version should be even more closely aligned.

Future amendments of the classification will be based on scientific advances. In some areas, pathology may be well described, but the understanding of cause and consequence may change rapidly. We believe placental pathologies may be such an area that should be followed closely. In other areas, we must still expect to knowingly choose sub-optimal classification for many years to come. One such problematic area is the traditional segregation of congenital anomalies from other fetal conditions, and the customary organ-system arrangement challenged by multiple organ anomalies. The close associations between single gene mutations and multiple organ defects (e.g. Smith-Lemli-Opitz syndrome[30]) further questions the prospective utility of classifying the resulting syndromes rather than causes of inductive or disruptive effects. The rationale may also seem weak for classifying single gene defects e.g. in metabolism as fetal metabolic disease if the defect is not associated with malformations, and as congenital anomalies if malformations develop because of the defect (again e.g. Smith-Lemli-Opitz syndrome). Three arguments can be made for coding congenital anomalies separately, and not with fetal conditions based on chromosomal, genetic, environmental or interacting causes: 1) Current knowledge would rarely allow for the correct classification. 2) Requirements to seek detailed genetic information would further skew coding by available resources for examinations. 3) Strengthened registration of major anomalies is needed to understand mortality and prevention in areas where this is not routinely performed.

## Conclusion

As all other systems, Codac is created to deliver the results it was designed for – there will never be a perfect system for all imaginable purposes. However, in repeated testing in different settings, the appropriately applied Codac system promises to better manage information on causes of perinatal deaths, the conditions associated with them, and the most common clinical scenarios for future study and comparisons.

## Abbreviations

COD: Cause of death; AC: Associated condition; ICD: International Classification of Diseases; PPROM: Preterm prelabor rupture of the membranes; SGA: Small for gestational age; WHO: World Health Organization.

## Competing interests

The authors declare that they have no competing interests.

## Authors' contributions

JFF lead the development of the original Codac system in consultation with HP, oversaw the conduct of the study and revision of the Codac system, and drafted the manuscript. VF oversaw the testing of the Codac system and analyzed the results with KG. SJG cross-referenced the revised Codac system to ICD-10 in consultation with JFF. CWD, FF, RCF, SJG and GCCS critically reviewed the revised version of Codac and the scientific content of the manuscript. All other investigators formed the classification teams and undertook review of stillbirth cases and data collection. All authors interpreted results, edited drafts of the manuscript and electronic Codac system, and read and approved the final manuscript.

## Pre-publication history

The pre-publication history for this paper can be accessed here:



## Supplementary Material

Additional File 1 **CODAC_II**. This is the Excel file that contains the actual classification system including interactive functions both to simplify classification as well as to provide automatic analysis and cross-reference to ICD-10. Macros must be allowed to enable the interactive functions. Further instructions on its use are found in the file itself. Click here for file
